# The relationship between three subtypes of safety behaviors and social anxiety: Serial mediating effects of state and trait post-event processing

**DOI:** 10.3389/fpsyg.2022.987426

**Published:** 2022-11-02

**Authors:** Dahye Kim, Ha-Yeon Kang, Jung-Kwang Ahn

**Affiliations:** Department of Psychology, Chungbuk National University, Cheongju, South Korea

**Keywords:** social anxiety, safety behaviors, impression management, avoidance behavior, anxiety-symptom control, post-event processing, serial mediation

## Abstract

The cognitive model for social anxiety disorder (SAD) highlights the role of safety behaviors and post-event processing (PEP). We identify the serial mediating effect of state and trait PEP between three types of safety behaviors (impression management, avoidance behavior, and anxiety-symptom control) and social anxiety. Given that the associations between the three subtypes of safety behaviors and two perspectives of PEP have not yet been examined, we aimed to investigate these relationships according to the level of social anxiety. A total of 487 participants participated in an online survey. Participants were classified into two groups, high and low, based on their social anxiety scores. We used Social Behavior Questionnaire to distinguish three types of safety behaviors and the State and Trait versions of the Post-Event Processing Inventory to identify two perspectives of PEP. We used descriptive statistics and an independent *t*-test to compare the high and low social anxiety groups. Mediation effects were examined using mediation analysis and bootstrapping with 5,000 replications. The results showed that the three safety behaviors had different effects on social anxiety *via* PEP. In the high social anxiety group, avoidance behavior and anxiety-symptom control predicted social anxiety positively, whereas impression management did not. However, with state PEP and trait PEP as mediators, impression management and avoidance behavior positively predicted social anxiety but not anxiety-symptom control. In the low social anxiety group, only avoidance behavior was significantly related to social anxiety, but when the state and trait PEP were mediated, the effect disappeared. These results indicated that impression management could affect social anxiety only when mediated by PEP in people with high social anxiety. A better understanding of the content and processes underpinning safety behavior and PEPs might have important implications for the prevention and treatment of social anxiety disorder.

## Introduction

Safety behaviors are mental processes and behavioral strategies used by individuals with social anxiety disorder (SAD) to reduce distress and suppress anxiety symptoms in fear-inducing social situations. Individuals with SAD believe that safety behaviors are necessary because they momentarily increase their sense of security (McManus et al., 2008). However, safety behaviors maintain SAD for several reasons. First, individuals with SAD cannot confirm that the catastrophes they fear will not occur if they do not use safety behaviors (Clark and Wells, 1995). They tend to believe that negative social consequences do not occur because they use safety behaviors (Salkovskis, 1991; Alden and Bieling, 1998), preventing them from realizing that their maladaptive beliefs about social situations are untrue (Makkar and Grisham, 2011). Second, safety behaviors themselves may create a bad impression, resulting in a negative evaluation from others (Wells et al., 1995). Third, safety behaviors used in social situations eventually lead to escaping from these situations, and thus, social anxiety symptoms persist because afflicted individuals do not experience a decrease in anxiety over time (Clark and Wells, 1995; Wells et al., 1995; McManus et al., 2008). Lastly, using safety behaviors in social situations is likely to induce post-event processing (PEP; Helbig-Lang et al., 2016; Piccirillo et al., 2016; Carnahan et al., 2020). PEP is an information processing method that monitors the negative self-perception and reactions of others in detail after a social situation (Hofmann, 2007). During this process, PEP makes it possible to retrieve past events that individuals with SAD consider to be failures, which then become encoded more negatively in the memory (Clark and Wells, 1995). Individuals with social anxiety engage in more negatively biased PEP, which in turn aggravates SAD (Dannahy and Stopa, 2007). Hofmann’s (2007) comprehensive model of SAD suggests that using safety behaviors during an anxiety-provoking social situation can lead to PEP and explicitly highlights the direct connection between the two variables.

Clark and Wells (1995) stated that safety behaviors consist of impression management and avoidance behaviors. Impression management is an attempt to feign a good impression on others by tightly monitoring and controlling one’s behaviors (Hirsch et al., 2004; Plasencia et al., 2011). Those who use this strategy attempt to self-monitor excessively, rehearse too much, and speak only perfectly appropriate words (Hirsch et al., 2004; Plasencia et al., 2011). Avoidance behaviors are defined as low self-disclosure and hiding oneself in social situations (Plasencia et al., 2011). For example, speaking less in the presence of others and avoiding eye contact are frequently observed avoidance behaviors (Clark and Wells, 1995). Recently, a new subtype of safety behavior, called anxiety-symptom control, was identified (Evans et al., 2021; Kim and Ahn, 2021), which is similar to the subtype that involves hiding the physical symptoms of anxiety. Physical anxiety symptoms, as suggested by Cuming et al. (2009), are about hiding only visible symptoms of the autonomic nervous system. Anxiety-symptom control involves not only physical symptoms (e.g., using makeup to hide blushing; gripping a glass tightly to hide trembling) but also cognitive efforts (e.g., blanking out or switching off mentally; avoiding pauses in speech) in dealing with anxiety (Kim and Ahn, 2021).

These subtypes of safety behaviors have different effects on social anxiety. Avoidance behavior is related to high state anxiety levels, which maintain social anxiety (Hirsch et al., 2004; Sparrevohn and Rapee, 2009). Individuals who display these behaviors are negatively evaluated by others and not preferred as conversation partners (Plasencia et al., 2011; Gray et al., 2019). Anxiety-symptom control appears to be related to self-focused attention (Hofmann, 2007; McManus et al., 2008) which involves concentrating on physical anxiety symptoms and negative perceptions of oneself (Clark, 2005), making it difficult to perceive and assign attention to external information thereby reducing performance in social situations (Spurr and Stopa, 2002). Evans et al. (2021) suggested that the effects of impression management on social anxiety may differ from those of other safety behaviors. Unlike avoidance safety behavior, those who used impression management were rated more positively, as less anxious, and more likable by conversation partners (Hirsch et al., 2004; Plasencia et al., 2011; Gray et al., 2019). In addition, people engaging in impression management safety behaviors did not think that they looked anxious (Hirsch et al., 2004; Gray et al., 2019). Anxiety-symptom control and avoidance behaviors are highly correlated with social anxiety. However, impression management shows only a moderate correlation, displaying somewhat different characteristics from the other two (Plasencia et al., 2011; Kim and Ahn, 2021). Despite these characteristics, impression management, like the other safety behaviors, prevents socially anxious individuals from facing social situations and learning that the anxiety decreases over time (Clark and Wells, 1995; Wells et al., 1995). Furthermore, impression management is related to cost predictions for future social events (Plasencia et al., 2011). People with SAD who use impression management find it difficult to endure social situations without this strategy. They have a deep-rooted fear of losing control over their behavior in future social situations and being unable to make a good impression. Impression management may differ from the other two safety behaviors in having some advantages in social interactions; nevertheless, it maintains SAD. Considering the dual nature of impression management, we speculated that the association between impression management and social anxiety might be suppressed (inconsistent mediation; MacKinnon et al., 2000; Shrout and Bolger, 2002). People who use the impression management technique do not anticipate being negatively evaluated because they do not think they would not look anxious. Thus, their social anxiety may decrease in the short term (Hirsch et al., 2004; Gray et al., 2019). However, in the long run, people who use impression management would experience PEP after a social situation because they are unable to confirm that their negative social beliefs (e.g., “Negative social consequences did not happen because I had tried to come across well.”) are incorrect (Mitchell and Schmidt, 2014). Therefore, social anxiety would also be maintained in those who use impression management through the experiencing of PEP.

PEP can be considered from two perspectives: state and trait (Brozovich and Heimberg, 2011; Blackie and Kocovski, 2017). State perspective of PEP is related to the situation-specific context, and trait PEP is the general tendency to engage in PEP (Blackie and Kocovski, 2017). State PEP is provoked by social situations where anxiety and fear are felt (Maeda et al., 2021). Trait PEP is a general, relatively stable tendency to engage in post-event thought on anxiety-provoking social situations (Blackie and Kocovski, 2017; Maeda et al., 2021). State PEP tends to strengthen negative self-perceptions in people with social anxiety (Clark, 2005). Reviewing social situations through negatively biased self-perceptions during state PEP will influence trait PEP in other social situations (Hofmann, 2007). Thus, if state PEP is repeatedly triggered, it could affect the general tendency to engage in PEP (trait PEP). For example, if individuals with SAD experience PEP frequently or intensively in various social situations, this would persist and stabilize, and finally, transform into a trait.

Several studies have found that safety behaviors are likely to provoke PEP. According to Hofmann (2007), people with social anxiety anticipate mistakes in social situations and use safety behaviors to prevent them. Safety behaviors lead to PEP, creating a cycle that perpetuates SAD. In a study on university students, Carnahan et al. (2020) confirmed that the higher the level of social anxiety, the more the safety behaviors used, which in turn increases PEP. Mitchell and Schmidt (2014) studied the contribution of safety behavior to PEP in people with high social anxiety levels and found that, both immediately after the experimental event and 4 days later, PEP could be related to safety behavior. However, these studies did not consider the effects of the subtypes of safety behaviors on PEP, or the two perspectives of PEP. In addition, there are differences in the types of safety behaviors primarily used and the frequency of safety behaviors between people with high and low levels of social anxiety (McManus et al., 2008), which in turn, can lead to different intensities and frequencies of PEP. Therefore, this study aimed to identify a serial mediating model with PEP as a mediator in the relationship between safety behaviors and social anxiety by classifying participants into low and high social anxiety groups.

Given that the associations between the three subtypes of safety behaviors and the two perspectives of PEP have not been empirically examined, the goal of our study was to investigate these relationships according to participants’ level of social anxiety. First, we expected that avoidance behavior and anxiety-symptom control would be positively associated with social anxiety, but impression management may negatively predict social anxiety in the high social anxiety (high SA) group. This pattern might not appear in the low social anxiety (low SA) group. Second, we tested a preliminary cross-sectional hypothesis that state and trait PEP would mediate the relationship between safety behaviors and social anxiety symptoms in the high SA group but not in the low SA group. Third, this study aimed to explore the negative association between impression management and social anxiety in the high SA group that could be suppressed and turned into a positive association by state and trait PEP (inconsistent mediation).

## Materials and methods

Participants were recruited for the online survey through a research company. Inclusion criteria for the participants were (1) age between 19 and 59 years and (2) understanding of the Korean language. The age criterion was established considering that people seeking treatment for anxiety disorders in Korean clinical settings are mainly between the ages of 19 and 59 years (Korea Ministry of Health and Welfare, 2019). Data from 1,924 individuals were collected; a total of 1,182 participants were excluded because they did not fulfill the age criterion (*n* = 777), dropped out (*n* = 350), or provided careless responses (*n* = 55). We divided the participants into the high SA and the low SA groups. Participants were categorized based on mean scores on the Social Interaction Anxiety Scale-6 and Social Phobia Scale-6 (SIAPS-12). Participants were classified into the high SA group if they scored 21 (approximately +1 standard deviation) or higher on the SIAPS-12—a well-established cut-off score associated with SAD in Korean samples (Kim et al., 2013). When dividing the groups by a cut-off point, it was problematic to accept participants with a high score below the cut-off (e.g., 20 or 19 points) as representative of low SA. Thus, participants who scored 7 or under (−1 standard deviation from the mean) on the SIAPS-12 were classified into the low SA group. This categorization could better represent high and low SA groups. Finally, 487 individuals (260 high SA group, 227 low SA group) were analyzed in this study. The data from this study were also used in the validation of the Korean versions of the Social Behavior Questionnaire (SBQ) and the Post-Event Processing Inventory (PEPI).

### Measures

#### SBQ

The SBQ is a 28-item self-report measure used to assess the frequency of safety behaviors related to social anxiety. Clark et al. (1995) developed this scale, while Kim and Ahn (2021) validated the Korean version, which consists of impression management (e.g., “Make an effort to come across well”), avoidance behavior (e.g., “Talk less”) and anxiety-symptoms control (e.g., “Wear clothes or makeup to hide blushing”). The items are rated on a Likert-scale ranging from 0 (never) to 3 (always). A higher score indicates the use of more safety behaviors. All subscales demonstrated good internal consistency (Total: Cronbach’s *α* = 0.92, impression management: Cronbach’s *α* = 0.89, avoidance behavior: Cronbach’s *α* = 0.87, anxiety-symptom control: Cronbach’s *α* = 0.82).

#### State and trait versions of the PEPI

The PEPI is a self-report measure developed by Blackie and Kocovski (2017), and it measures state (PEPI-S) and trait PEP (PEPI-T). The Korean version of the PEPI was validated by Pyo and Ahn (2021). The original versions of the PEPI-S and PEPI-T have 12 items each across three subscales: intensity, frequency, and self-judgment. However, their Korean versions consist of 10 items each and have two subscales: dysfunctional information processing (frequency: e.g., “I thought about the mistakes I made during the event,” “After social events, I think about the mistakes I made during the event”) and impairment in daily life (intensity: e.g., “My thoughts about the event interfered with my ability to concentrate,” “After social situations, my thoughts about the event interfere with my ability to concentrate”). Each item is rated on a Likert-scale ranging from 1 (strongly disagree) to 5 (strongly agree). A higher score indicates a higher level of PEP. The measure demonstrated excellent internal consistency (PEPI-S total: Cronbach’s *α* = 0.97; PEPI-T total: Cronbach’s *α* = 0.96).

#### SIAPS-12

The SIAPS is a 12-item self-report measure used to assess social anxiety symptoms. This is a short version of the Social Interaction Anxiety Scale (SIAS) and Social Phobia Scale (SPS). Originally, the two scales consisted of 20 questions each and were used together (Mattick and Clarke, 1998), but 40 items were considered to be a possible burden to participants (Jepson et al., 2005). Therefore, Peters et al. (2012) developed a shorter version of these scales to properly assess social anxiety symptoms using fewer items. Kim et al. (2013) validated the Korean version. This scale is used to distinguish between people with and without SAD. The Korean version of this measure consists of social interaction anxiety (e.g., “I find difficulty mixing comfortably with the people I work with”) and performance anxiety (e.g., “I get nervous that people are staring at me as I walk down the street”). The items are rated on a Likert scale ranging from 0 (not at all) to 4 (extremely). We summed the scores of the SIAS-6 and SPS-6 to determine the degree of social anxiety. A higher score indicates higher social interaction or performance anxiety. The SIAPS showed excellent internal consistency (total: Cronbach’s *α* = 0.96, SIAS-6: Cronbach’s *α* = 0.93, SPS-6: Cronbach’s *α* = 0.94).

### Statistical analysis

All analyses were conducted using the statistical software jamovi 2.3.13.0, based on the R statistical language (The jamovi project, 2022). First, descriptive statistics were used to confirm the demographics and characteristics of the major variables. Second, an independent sample *t*-test and *χ*^2^ test were performed to check whether there were differences in gender, age, region, job, years of education, and major variables between the two groups. When the equal-variance assumption was not satisfied, the Welch-Aspin test was performed (Ryan et al., 2012). The interpretation of the effect size is as follows: 0.2 is small, 0.5 is medium, 0.8 is large, 1.2 is very large, and 2.0 is huge (Cohen, 1988; Sawilowsky, 2009). Third, the associations between safety behavior, social anxiety, state PEP, and trait PEP were investigated using Pearson’s correlation analysis, following Cohen (1988) in interpreting the scale of effect size: 0.1 is small, 0.3 is medium, and 0.5 is large. Finally, to examine the mediating role of the state and trait PEP on the relationship between safety behaviors (impression management, avoidance behavior, and anxiety-symptom control) and social anxiety, jamovi’s Advanced Mediation Models (jAMM) analyses were performed (Gallucci, 2020). The *z*-score was calculated by dividing the mediation effect by the standard error. This value was compared against a standard normal distribution to test for significance. If the *z*-score was >1.96, we concluded that the effect is larger than would be expected by chance and considered the effect significant (Yay, 2017). The *z*-test associated with the mediated effect is the large sample *z*-test, which is a slightly more accurate version of the Sobel test (Gallucci, 2020). We used the bootstrapping method to test for the mediation effect. This method can be used even when the data do not meet the normality assumption (Shrout and Bolger, 2002). The bootstrapping method generated 5,000 samples to obtain 95% confidence intervals for indirect effects (Hayes and Rockwood, 2017). If the 95% confidence interval calculated using the bias-corrected bootstrapping method does not contain zero, the path coefficient is considered significant (Hayes and Rockwood, 2017). The significance level was set at *p* < 0.05.

## Results

### Demographics

Participant ranged from 19 to 59 years (*M* = 39.58, SD = 10.79). Among the participants, 239 (49.08%) were women. Table 1 shows the participants’ demographic characteristics.

**Table 1:** Demographic characteristics.

	*M* (SD)			*t/χ* ^2^
	Total (*N* = 487)	High SA (*n* = 260)	Low SA (*n* = 227)	
Age [range]	39.58 (10.79)	38.65 (10.73)	40.65 (10.79)	2.05^*^
Gender (*n*, %)				0.91^a^
Men	248 (50.92%)	133 (51.15%)	115 (50.66%)	
Women	239 (49.08%)	127 (48.85%)	112 (49.34%)	
Education (years completed)	15.23 (2.03)	15.12 (1.95)	15.36 (2.11)	1.29
Job (*n*, %)				0.29^a^
Office worker	185 (37.99%)	98 (37.69%)	87 (38.33%)	
Homemaker	52 (10.68%)	21 (8.08%)	31 (13.66%)	
Specialized job	46 (9.45%)	24 (9.23%)	22 (9.69%)	
Students	44 (9.03%)	24 (9.23%)	20 (8.81%)	
Production, technology, and labor	26 (5.34%)	13 (5.00%)	13 (5.73%)	
Service, sales, and sales job	26 (5.34%)	16 (6.15%)	10 (49.41%)	
Freelancer	25 (5.13%)	10 (3.85%)	15 (6.61%)	
Inoccupation	24 (4.93%)	14 (5.38%)	10 (4.41%)	
Public official	19 (3.90%)	14 (5.38%)	5 (2.20%)	
Teacher and academy lecturer	19 (3.90%)	14 (5.38%)	5 (2.20%)	
Self-employment	11 (2.26%)	7 (2.69%)	4 (1.76%)	
Management	6 (1.23%)	4 (1.54%)	2 (0.88%)	
Agriculture, forestry, and fishing	3 (0.62%)	1 (0.38%)	2 (0.88%)	
Others	1 (0.21%)	0 (0.00%)	1 (0.44%)	

Age and education (numbers in) are standard deviations.

a *χ*^2^ score.

* *p* < 0.05.

### Group differences In safety behaviors, PEP, and social anxiety

Table 2 presents the differences and descriptive statistics for the major variables between the two groups. In the low SA group, the level of social anxiety was very low (*M* = 2.32, SD = 2.42), and the score of impression management was higher than other safety behaviors (*M* = 13.22, SD = 7.01). There were significant differences in all variables between the two groups, all *p*s < 0.001. The difference in social anxiety indicated a huge effect size, *t*(361.88) = 64.81, *p* < 0.001 *d* = 5.75, but impression management showed medium to large size difference, *t*(414.16) = 7.32, *p* < 0.001 *d* = 0.67. The rest had very large to huge differences, avoidance behavior: *t*(485) = 17.91, *p* < 0.001, *d* = 1.63; anxiety-symptom control: *t*(445.23) = 18.99, *p* < 0.001, *d* = 1.70.

**Table 2:** Group differences in the major variables.

	*M* (SD)			***t***	Cohen’s ***d***
	Total	High SA	Low SA		
	(*N* = 487)	(*n* = 260)	(*n* = 227)		
Impression management	15.44 (6.46)	17.37 (5.24)	13.22 (7.01)	7.32^***^	0.67
Avoidance behavior	9.04 (5.08)	12.03 (4.15)	5.61(3.70)	17.91^***^	1.63
Anxiety-symptom control	7.64 (4.94)	10.60 (4.46)	4.25 (2.84)	18.99^***^	1.70
State PEP	27.42 (10.12)	33.57 (5.94)	20.38 (9.31)	18.87^***^	1.69
Trait PEP	27.11 (9.83)	33.40 (5.79)	19.90 (8.47)	20.75^***^	1.86
Social anxiety	15.63 (13.21)	27.24 (5.63)	2.32 (2.42)	64.81^***^	5.75

PEP, Post-Event Processing; SA, Social Anxiety; The *t*-test of avoidance behavior is the result of Student’s *t*-test, and the others are the results of Welch-Aspin test. ***p < 0.001.

### Correlational analysis

Table 3 shows correlations for the major variables in each group. All variables were positively correlated, and significant positive correlations were found between all safety behaviors and social anxiety in both groups. In the high SA group, impression management showed a medium effect size with social anxiety alone, *r* = 0.32 *p* < 0.001, while other safety behaviors had medium to large correlations. All safety behaviors had significant positive correlations with PEPI-S and PEPI-T, indicating that a higher level of any of the safety behaviors is related to higher PEPI-S and PEPI-T.

**Table 3:** Correlations between the variables.

	1	2	3	4	5	6
1. Impression management	–	0.54^***^	0.40^***^	0.47^***^	0.46^***^	0.32^***^
2. Avoidance behavior	0.51^***^	–	0.42^***^	0.45^***^	0.47^***^	0.40^***^
3. Anxiety-symptom control	0.59^***^	0.51^***^	–	0.30^***^	0.28^***^	0.47^***^
4. State PEP	0.30^***^	0.25^***^	0.31^***^	–	0.89^***^	0.46^***^
5. Trait PEP	0.32^***^	0.26^***^	0.31^***^	0.86^***^	–	0.32^***^
6. Social anxiety	0.20^**^	0.29^***^	0.24^***^	0.38^***^	0.24^***^	–

PEP, Post-Event Processing. The lower part of the diagonal is the result of the low SA group and the upper part of the diagonal is the result of the high SA group.

** *p* < 0.01;

*** *p* < 0.001.

### Mediating effects

**Figure 1:**
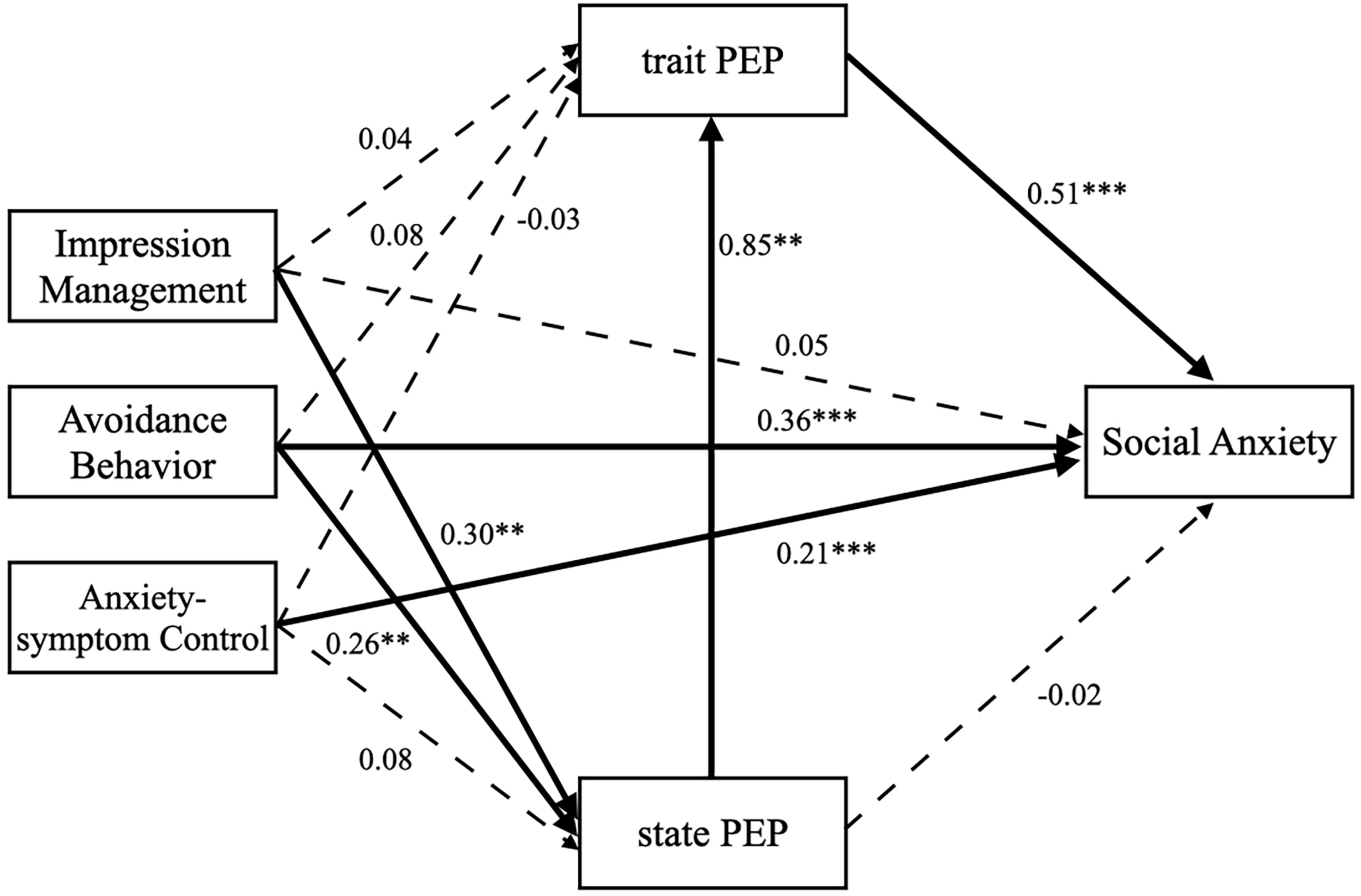
The serial mediation effects between the three subtypes of safety behavior and social anxiety in high SA group. The dotted lines represent non-significant paths, and the solid lines represent significant paths, ^**^p < 0.01 and ^***^p < 0.001.

**Figure 2:**
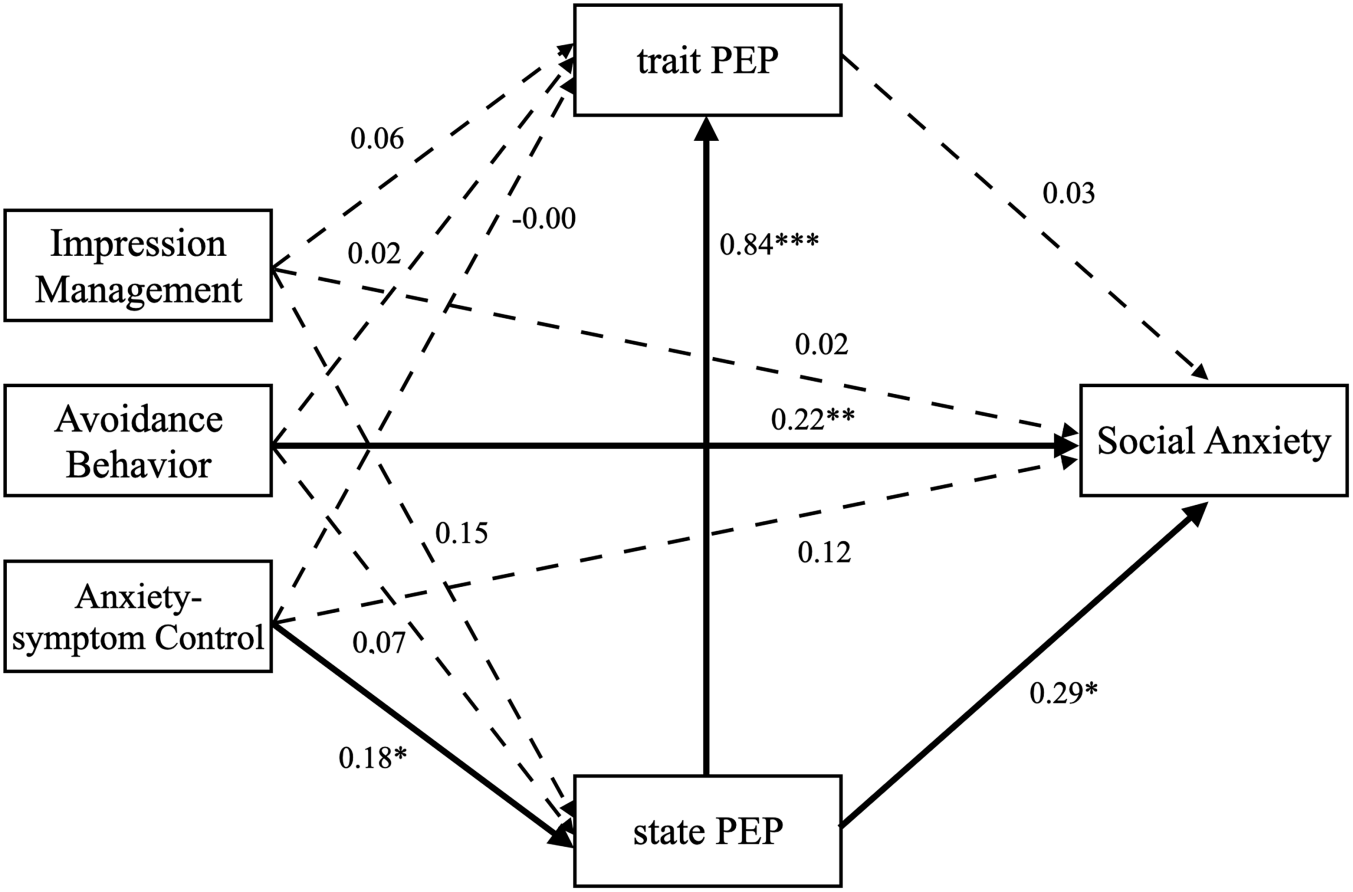
The serial mediation effects between the three subtypes of behavior and social anxiety in low SA group. The dotted lines represent non-significant paths, and the solid lines represent significant paths, ^*^p < 0.05, ^**^p < 0.01 and ^***^p < 0.001.

Table 4 presents the results of the mediation analysis for safety behaviors and social anxiety. Figures 1, 2 indicate the results of the high and low SA groups, respectively. In the high SA group, avoidance behavior and anxiety-symptom control predicted social anxiety significantly, *β* = 0.36, *p* < 0.001; *β* = 0.21, *p* < 0.001. However, impression management did not predict social anxiety significantly, *β* = 0.05, *p* = 0.448. PEP-S and PEP-T did not significantly mediate the relationship between a particular subtype of safety behavior and social anxiety, all *p*s > 0.05. However, the relationships between impression management and social anxiety and between avoidance behavior and social anxiety were positively significant when PEPI-S and PEPI-T mediated these relationships serially, *β* = 0.13, *p* = 0.041; *β* = 0.11, *p* = 0.005. Anxiety-symptom control did not predict social anxiety significantly when serially mediated by PEPI-S and PEPI-T, *β* = 0.03, *p* = 0.246.

**Table 4:** Total, direct, and indirect effects on the relationship between the subtypes of safety behaviors and social anxiety.

	Effect	High SA (*n* = 260)						Low SA (*n* = 227)					
		Estimate	SE	95% CI		*β*	*z*	Estimate	SE	95% CI		*β*	*z*
				Lower	Upper					Lower	Upper		
Total	IM → SA	0.05	0.07	−0.08	0.19	0.05	0.76	0.01	0.03	−0.05	0.06	0.02	0.20
	AB → SA	**0.48**	**0.09**	**0.31**	**0.66**	0.36^***^	**5.42**	**0.14**	**0.05**	**0.04**	**0.24**	0.22^**^	**2.81**
	AC → SA	**0.27**	**0.08**	**0.12**	**0.42**	0.21^***^	**3.52**	0.11	0.07	−0.03	0.24	0.12	1.49
Direct	IM → SA	−0.10	0.10	−0.28	0.09	−0.09	−1.04	−0.01	0.03	−0.07	0.04	−0.03	−0.41
	AB → SA	**0.28**	**0.11**	**0.06**	**0.49**	0.21^*^	**2.53**	**0.13**	**0.05**	**0.04**	**0.22**	0.19^**^	**2.65**
	AC → SA	**0.25**	**0.07**	**0.11**	**0.38**	0.20^***^	**3.59**	0.05	0.07	−0.08	0.19	0.06	0.79
Indirect	IM → PEP-T → SA	0.02	0.03	−0.03	0.07	0.02	0.75	0.00	0.00	−0.00	0.01	0.00	0.22
	IM → PEP-S → SA	−0.01	0.05	−0.14	0.08	−0.00	−0.09	0.02	0.01	0.00	0.05	0.04	1.35
	AB → PEP-T → SA	0.06	0.05	−0.01	0.18	0.04	1.24	0.00	0.00	-0.01	0.01	0.00	0.09
	AB → PEP-S → SA	−0.01	0.06	−0.12	0.12	−0.00	−0.09	0.01	0.02	−0.01	0.06	0.02	0.76
	AC → PEP-T → SA	−0.02	0.02	−0.06	0.02	−0.01	−0.96	−0.00	0.01	−0.01	0.01	−0.00	−0.00
	AC → PEP-S → SA	−0.00	0.02	−0.06	0.03	−0.00	−0.08	0.05	0.03	0.00	0.13	0.05	1.49
	IM → PEP-S → PEP-T → SA	**0.14**	**0.07**	**0.04**	**0.31**	0.13^*^	**2.04**	0.00	0.01	−0.01	0.02	0.00	0.22
	AB → PEP-S → PEP-T → SA	**0.15**	**0.05**	**0.07**	**0.30**	0.11^**^	**2.79**	0.00	0.01	−0.01	0.03	0.00	0.16
	AC → PEP-S → PEP-T → SA	0.04	0.04	−0.01	0.13	0.03	1.16	0.00	0.02	−0.03	0.05	0.01	0.23

Confidence intervals computed with method: Bias corrected bootstrap, Betas are completely standardized effect sizes, z = mediated effect divided by its standard error, Statistically significant values are bold typeface. SA, Social Anxiety; IM, Impression Management; AB, Avoidance Behavior; AC, Anxiety-symptom Control; PEP-S, Post-Event Processing-State; PEP-T, Post-Event Processing-Trait.

* *p* < 0.05;

** *p* < 0.01;

*** *p* < 0.001.

In the low SA group, avoidance behavior also predicted social anxiety significantly and positively like in the high SA group, *β* = 0.22, *p* = 0.005, but impression management and anxiety-symptom control did not predict social anxiety significantly, *β* = 0.02, *p* = 0.842; *β* = 0.12, *p* = 0.135. PEPI-S and PEPI-T were not significant mediators in all relationships between safety behaviors and social anxiety, and they did not mediate these relationships sequentially, all *p*s > 0.05.

## Discussion

The aim of the present study was to identify the relationship between the subtypes of safety behavior and social anxiety mediated by state and trait PEP in high and low SA groups using a serial mediation model. In the high SA group, avoidance behavior and anxiety-symptom control predicted social anxiety positively, whereas impression management did not predict it. However, when state PEP and trait PEP were mediators, impression management and avoidance behavior positively predicted social anxiety, but not anxiety-symptom control. In the low SA group, impression management and anxiety-symptom control of safety behaviors did not predict social anxiety significantly. Only avoidance behavior significantly predicted social anxiety, and this disappeared when mediating state and trait PEP.

There was a difference in social anxiety scores and safety behavior scores between the two groups. In impression management, the effect size of the difference between the two groups was smaller than those of the other two safety behaviors. Given that impression management scores were higher than avoidance behaviors and anxiety-symptom controls in the low SA group, this implies that impression management was often used in the low SA group, unlike other safety behaviors. Correlation analysis results indicated that all three types of safety behaviors were positively related to social anxiety in both groups. It suggests that the use of safety behaviors is associated with social anxiety regardless of the level of social anxiety symptoms. However, in impression management, the effect size of the correlation was smaller than that of the other safety behaviors in both groups. This might be because impression management behaviors, such as trying use the appropriate words out accurately and thinking positively, are what most people use to behave appropriately in social situations. On the other hand, the high SA group might use impression management strategies to deal with anxiety or fear of negative evaluations. Impression management used for this purpose seem to function as a safety behavior and maintains their social anxiety.

According to our model, the relationship between safety behaviors and social anxiety in high and low SA is different. Primarily, in the high SA group, the total effects of avoidance behavior and anxiety-symptom control on social anxiety were positive, but impression management was not associated with social anxiety. This indicates that avoidance behavior and anxiety-symptom control use could be associated with increased social anxiety. Avoidance behavior is a strong negative reinforcement that influences the maintenance of SAD (Rachman et al., 2000). It prevents people with social anxiety from learning that it may not be as dangerous as they think to be exposed to social situations that they fear. Anxiety-symptom control is highly related to self-focused attention, which maintains SAD (Clark and Wells, 1995; Hofmann, 2007; Kim and Ahn, 2021). Focusing attention inward to deal with anxiety symptoms reduces social performance and makes it challenging to process others’ reactions objectively (Spurr and Stopa, 2002). In addition, self-focused attention increases anxiety symptoms, confirming negative self-images (Clark and Wells, 1995). In sum, we suggest that both avoidance behavior and anxiety-symptom control have adverse effects on social anxiety. However, according to our model, impression management is not associated with social anxiety after controlling the other two safety behaviors. This is consistent with the results of previous studies that the other two types of safety behaviors negatively affect social anxiety, whereas impression management has slightly different characteristics (Hirsch et al., 2004; Plasencia et al., 2011; Gray et al., 2019; Evans et al., 2021). The other two safety behaviors are clearly related to avoidance, but impression management has more subtle avoidance behaviors. In addition, impression management is perceived positively by others and overlaps with general impression management which is independent of safety behavior, such that the relationship with safety behavior may not be clear. Moreover, impression management might not affect social anxiety in the short term because it helps the individual appear less anxious than the other two safety behaviors. In contrast, only the path from avoidance behavior to social anxiety was significant in the low SA group. Although the overall symptoms of the low SA group were low, avoidance behavior was still related to social anxiety. Even after receiving successful cognitive behavioral therapy, people who avoid social situations can easily experience a recurrence of their social anxiety symptoms (van Uijen et al., 2018). Considering this, we think that avoidance behaviors might be one of the worst risk factors for the relapse of SAD. Therefore, it is suggested that therapists treating SAD should focus more on eliminating avoidance behaviors.

Impression management was related to increased social anxiety when state and trait PEP were induced. In the high SA group, impression management and avoidance behavior were associated with social anxiety by the mediating effects of state and trait PEP, but anxiety-symptom control was not significantly related. Impression management was not directly related to social anxiety, but it was associated with social anxiety by the mediating effect of the two perspectives of PEP. Although it was not an inconsistent mediation, the relationship between impression management and social anxiety seemed to deteriorate through state and trait PEP. This result is in line with several prior findings that impression management maintains social anxiety as a safety behavior (Clark and Wells, 1995; Hirsch et al., 2004). Particularly, individuals who use impression management during social events cannot confirm that their negative beliefs about social situations are incorrect. Thus, individuals experience state PEP that could make them review their behavior and double whether they made a good impression. They then constantly review the information that fits their negative beliefs, which may lead to trait PEP. Persistent PEP can make individuals afraid of future social situations. Particularly, the use of impression management is related to the cost prediction of future social situations, which in turn may further increase anxiety about such situations (Plasencia et al., 2011). No matter how individuals try to make a good impression, and even if they do not get a negative evaluation from others, impression management can never be considered a helpful strategy because social anxiety is maintained when mediated by state and trait PEP.

In the high SA group, avoidance behavior is related to social anxiety, whether or not mediated by state PEP and trait PEP. Anxiety-symptom control, which was positively related to social anxiety, did not predict social anxiety significantly through state and trait PEP. This might be because of the contents of PEP in people with SAD. During PEP, individuals with SAD create negative images of themselves in social situations (Clark and Wells, 1995). These negative self-images are usually about impression management (e.g., “Did I speak the right word?” or “Did I look nervous?”), or avoidance behaviors (e.g., imagery of not being able to make eye contact or not having a conversation), and not anxiety-symptom control itself (e.g., “Did I grip cups or glasses tightly?” or “Did I wear clothes or make-up to hide blushing?”). In other words, the PEP is about the outcome of the anxiety-symptom control (e.g., How I looked to other people), not the contents of anxiety-symptom control themselves. In this way, impression management and avoidance behavior may trigger PEP, which can increase social anxiety, but anxiety-symptom control may not. Thus, when PEPs were the mediators, the relationship between anxiety-symptom control and social anxiety was not significant. In addition, anxiety-symptom control shares features of avoidance behavior (Kim and Ahn, 2021). Anxiety-symptom control includes hiding and controlling the physical symptoms of anxiety. Hiding physical symptoms is similar to avoidance behavior, often used as a safety behavior by people with social anxiety (Kim and Ahn, 2021). Therefore, the relationship between social anxiety and anxiety symptom control seems to decrease after controlling for avoidance behavior. Although the two safety behaviors look similar, it is still meaningful to distinguish them because they have different purposes and require different therapeutic approaches (Alden and Bieling, 1998; Sparrevohn and Rapee, 2009). However, research on anxiety-symptom control is insufficient, and much investigation is needed.

In the low SA group, all pathways were not significant when mediated by state and trait PEP. This means that the safety behaviors used in the low SA group do not have an effect on maintaining social anxiety. This may be because the low SA group did not experience PEP because the safety behavior was not used for the purpose of suppressing and dealing with social anxiety, or fewer safety behavior were used by them.

The present study has the following limitations. First, since this study was cross-sectional, future research on the relationship between safety behaviors and social anxiety using longitudinal data would be required. Second, we have only confirmed the mechanism of the relationship between safety behavior and PEP, and the causal relationship would need to be verified by an experimental study manipulating the safety behaviors. Third, to our knowledge, this is the first attempt to confirm the relationship between state/trait PEP and the three subtypes of safety behaviors. Therefore, further research, such as using ecological momentary assessment, is needed. Fourth, we did not include potential variables that could confound the model, such as recent life events that may influence state PEP or affective conditions. Future studies need to properly control for these variables to demonstrate the causal relationship between safety behavior and PEP. Finally, although we divided our participants into high and low SA groups, we did not control for other comorbid disorders such as depression and other anxiety disorders. Therefore, we recommend that future research replicate the model of this study for clinical populations.

Despite the limitations, this study confirms the trusted theoretical cognitive model (Hofmann, 2007) and several other previous studies on the relationship between safety behavior and PEP (Carnahan et al., 2020). Earlier studies have shown that using safety behaviors in clinical settings reduces the effectiveness of the treatment for SAD (Morgan and Raffle, 1999; Kim, 2005; van Uijen et al., 2018), but the reason has been inadequately explored. We offer an explanation for the maintenance mechanism of SAD by considering key associated variables in more detail. We propose that safety behaviors, especially impression management, could not increase social anxiety directly, and they require cognitive factors, such as PEP, to affect social anxiety.

## Data availability statement

The data analyzed in this study is subject to the following licenses/restrictions: The datasets generated and analyzed during the current study are not publicly available because we do not have the consent of the ethics committee or our participants to grant access to the collected data. However, they are available from the corresponding author on reasonable request. Requests to access these datasets should be directed to J-KA, jkahn@cbnu.ac.kr.

## Ethics statement

The studies involving human participants were reviewed and approved by Institutional Review Board in Chungbuk National University (CBNU-202021-HR-0206).

## Author contributions

J-KA authors developed the study concept and design. DK and H-YK conducted the data analysis and interpretation. DK drafted the paper under the supervision of J-KA. All authors contributed to the article and approved the submitted version.

## Funding

This work was supported by the research grant of the Chungbuk National University in 2019.

## Conflict of interest

The authors declare that the research was conducted in the absence of any commercial or financial relationships that could be construed as a potential conflict of interest.

## Publisher’s note

All claims expressed in this article are solely those of the authors and do not necessarily represent those of their affiliated organizations, or those of the publisher, the editors and the reviewers. Any product that may be evaluated in this article, or claim that may be made by its manufacturer, is not guaranteed or endorsed by the publisher.
